# Effect of the high-level trigger for detecting long-lived particles at LHCb

**DOI:** 10.3389/fdata.2022.1008737

**Published:** 2022-11-07

**Authors:** Lukas Calefice, Arthur Hennequin, Louis Henry, Brij Jashal, Diego Mendoza, Arantza Oyanguren, Izaac Sanderswood, Carlos Vázquez Sierra, Jiahui Zhuo, Part of LHCb-RTA Collaboration

**Affiliations:** ^1^Laboratoire de Physique Nucléaire et de Hautes Energies, Sorbonne Université, CNRS/IN2P3, Paris, France; ^2^Fakultät Physik, Technical University Dortmund, Dortmund, Germany; ^3^Laboratory for Nuclear Science, Massachusetts Institute of Technology, Cambridge, MA, United States; ^4^European Organization for Nuclear Research (CERN), Geneva, Switzerland; ^5^Instituto de Física Corpuscular (IFIC), Consejo Superior de Investigaciones Científicas, University of Valencia, Valencia, Spain

**Keywords:** LHCb, trigger, real time analysis, long-lived particles, GPU, SciFi, beyond standard physics

## Abstract

Long-lived particles (LLPs) show up in many extensions of the Standard Model, but they are challenging to search for with current detectors, due to their very displaced vertices. This study evaluated the ability of the trigger algorithms used in the Large Hadron Collider beauty (LHCb) experiment to detect long-lived particles and attempted to adapt them to enhance the sensitivity of this experiment to undiscovered long-lived particles. A model with a Higgs portal to a dark sector is tested, and the sensitivity reach is discussed. In the LHCb tracking system, the farthest tracking station from the collision point is the scintillating fiber tracker, the SciFi detector. One of the challenges in the track reconstruction is to deal with the large amount of and combinatorics of hits in the LHCb detector. A dedicated algorithm has been developed to cope with the large data output. When fully implemented, this algorithm would greatly increase the available statistics for any long-lived particle search in the forward region and would additionally improve the sensitivity of analyses dealing with Standard Model particles of large lifetime, such as $K_{\text{S}}^{0}$ or Λ^0^ hadrons.

## 1. Introduction

The Large Hadron Collider beauty (LHCb) (Alves et al., 2008) is one of the experiments based at the CERN Large Hadron Collider (LHC) that aims to explore the limitations of the well-tested Standard Model of particle physics. The imbalance of matter and antimatter in the Universe, the presence of neutrino masses, and the nature of dark matter are some examples of open questions for models of undiscovered physics beyond the Standard Model (BSM) that has been proposed.

The observation of the new processes that may be currently indiscernible is directly related to the accelerator luminosity. To meet the new challenges posed by Run 3 data taking at the LHC, the LHCb detector has undergone a major upgrade (LHCb, 2014a) to run at a higher luminosity of $L_{\text{inst}}=2\times 10^{33}\text{c}{\text{m}}^{-2}{\text{s}}^{-1}$. This luminosity corresponds to an averaged bunch collision frequency of 30 MHz, from where the full detector readout produces around 4 TB/s of raw data. To handle such detector occupancy and to search for rare processes among well-known SM events are computing challenges managed by the *trigger* system. The trigger system decides what information is recorded for later analysis. It must use simplified criteria to maintain a minimal latency. The previously used L0 hardware system is not suitable for such a high luminosity. Therefore, the LHCb trigger has been redesigned to remove the L0 hardware system and rely on a fully-software based architecture, which consists of two stages: HLT1 and HLT2 (LHCb, 2020), explained in the following sections.

## 2. Materials and methods

### 2.1. The LHCb detector

The upgraded LHCb detector, which will be operating during Run 3, detailed by Bediaga et al. (2012), is summarized below. The most important detector improvements as compared to the previous detector (Alves et al., 2008) are the new tracking system and the readout architecture. The readout architecture uses a purely software-based trigger that enables data to be recorded at five times the previous rate, and increases the acceptance of purely hadronic b decays by up to a factor of 2. LHCb is comprised of a tracking system (Vertex Locator, Upstream Tracker, and SciFi tracker), as well as two Ring Imaging Cherenkov detectors, a hadronic calorimeter, and an electromagnetic and muon chambers. These are summarized below.

**Figure 1:**
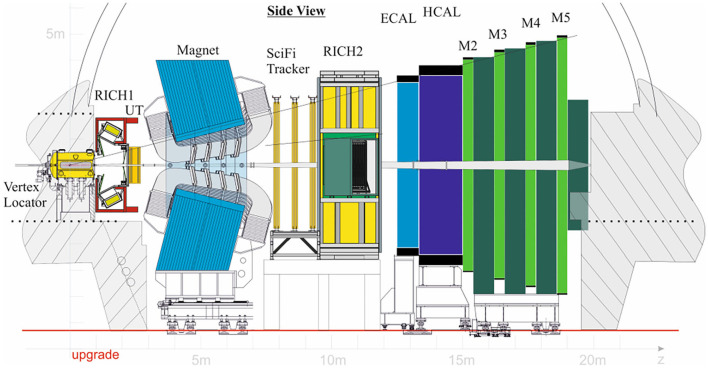
Sketch of the new LHCb detector.

The VErtex LOcator (VELO) is based on pixelated-silicon sensors and is critical to determine the decay vertices of b and c flavored hadrons. The Upstream Tracker, UT, contains vertically-segmented silicon strips and continues the tracking upstream of the VELO. It is also used to determine the momentum of charged particles with Δ*p*/*p* ≈ 10% and is useful for removing low-momentum tracks from being extrapolated downstream, thus speeding up the software trigger by about a factor of 3. Tracking after the magnet is handled by the new scintillating fiber-based Scintillating Fiber detector (SciFi). Two Ring Imaging CHerenkov (RICH) detectors supply particle identification. RICH1 is mainly for lower momentum particles and RICH2 is for higher momentum ones. The Electromagnetic Calorimeter (ECAL) identifies electrons and reconstructs photons and neutral pions. The Hadronic Calorimeter (HCAL) measures the energy deposits of hadrons, and the muon chambers M2-M5 are mostly used for muon identification. Figure 1 shows the LHCb upgrade detector.

### 2.2. The LHCb event-builder

**Figure 2:**
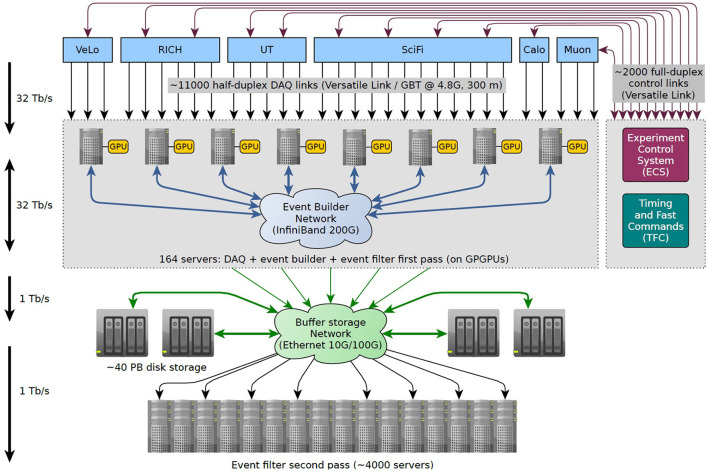
The architecture of the new LHCb data readout and event-buider system.

The main goal of the upgraded LHCb data-acquisition system is to enable the event filter to efficiently select events based on the full data readout from the detectors. The heart of the LHCb online system is the event builder, which is capable of collecting and aggregating data fragments coming from the FPGA front-ends of the various sub-detectors at the full collision rate of 30 MHz (LHCb, 2014b). The data size of a full event during the Run 3 is expected to be as high as 150 kB. Therefore, taking into account the data packed and the transfer protocol overheads, the new event builder has to sustain a throughput of up to 40 Tb/s. The event builder is essentially made up of three components: the readout units, the builder units, and the network interconnecting them. Each readout unit receives the event fragments from the detector point-to-point links and makes them available on the EB network. For each event, one of the builder units gathers all the event fragments from all the readout units, assembles them into a complete event, and writes it into HLT1 input buffer for event filtering. The present event-buider architecture relies on 163 EB servers interconnected through a 200 Gbits/s bi-directional network, as shown in Figure 2. The EB nodes have free PCIe slots that host GPU (Graphic Processing Units) cards to process the HLT1. This has the advantage of reducing the amount of data sent to the Event Filter Farm (EFF) and therefore reduces network costs.

### 2.3. Track types at LHCb

**Figure 3:**
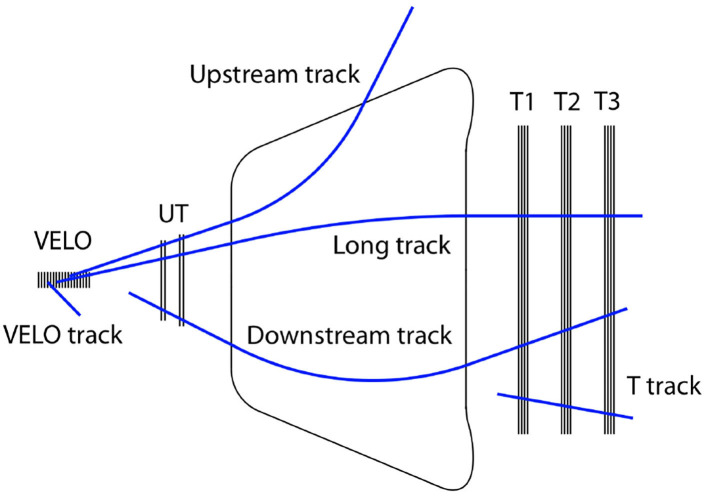
Track types in LHCb, as defined in LHCb (2014a).

In LHCb, the tracking system for charged particles consists of three subsystems: VELO, UT, and SciFi. The magnet is also necessary to bend particles to estimate their momentum. Several track types are defined depending on the subdetectors involved in the reconstruction, as shown in Figure 3.

The main track types considered for physics analyses are as follows:

- Long tracks, which have information from at least the VELO and the SciFi, and possibly the UT, are the main tracks used in physics analyses and at all stages of the trigger;
- Downstream tracks, which have information from the UT and the SciFi, but not VELO, typically correspond to decay products of $K_{\text{S}}^{0}$ and Λ^0^ hadron decays; and
- T-tracks, which only have hits from the SciFi, are typically not included either in the trigger or in analysis. Nevertheless, their potential for physics has been recently outlined (LHCb, 2022).

When simulating collision data, tracks meeting certain thresholds are defined to be reconstructible and have an assigned type based on the subdetector reconstructibility. This is based on the existence of reconstructed detector digits or clusters in the emulated detector, which are matched to the simulated particles if the detector hits they originated from are properly linked (Li et al., 2021). One can distinguish reconstructibility in the following subdetectors:

- VELO: at least 3 pixel sensors and at least 1 digit each.
- UT: at least 2 clusters where 1 cluster has to be in layer one or two and 1 in layer three or four. The clusters can be x or stereo clusters.
- SciFi: at least 1 x cluster and 1 stereo cluster in each of the 3 SciFi stations.

Requirements for reconstructible Long tracks implies VELO and SciFi reconstructibility, Downstream tracks must satisfy the UT and SciFi reconstructibility, and T-tracks only requires SciFi reconstrucibility.

### 2.4. The high-level trigger

The trigger of the LHCb detector readout in Run 3 and later is fully software based and comprises two levels: HLT1 and HLT2, described in LHCb (2020). Most notably, the HLT1 level has to be executed at a 30 MHz rate, which, thus, suffers from heavy constraints on timing for event reconstruction.

**Figure 4:**
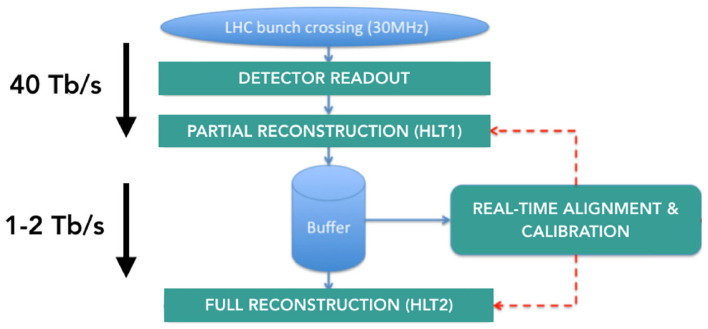
Dataflow for the first level of the trigger in the upgraded LHCb detector.

The HLT1 is the first trigger stage and performs partial event reconstruction to reduce the data rate. Tracking algorithms play a key role on fast event decisions, and the fact that they are inherently parallelizable processes suggests a way to increase the trigger performance. Thus, the HLT1 has been implemented on a number of GPUs through the Allen project (Aaij et al., 2020), which manages 4 TB/s and reduces the data rate by a factor of 30. After this initial selection, data are passed to a buffer system that allows real-time calibration and alignment of the detector. This is used for the full and improved event reconstruction carried out by HLT2. Figure 4 shows the dataflow at LHCb in the first trigger level.

Due to timing constraints, the current LHCb implementation in this stage is based on partial reconstruction and focuses solely on *long* tracks, i.e., tracks that have hits in the VELO. This trigger thus significantly affects the identification of particles with long lifetimes, in particular LLP searches in LHCb, where some of the final-state particles are created further than roughly a meter away from the IP and thus outside of the VELO acceptance.

Implementing new fast tracking algorithms in HLT1, allowing direct access to Downstream and T tracks from HLT1 selections, could solve this actual LHCb inability in LLPs reconstruction.

### 2.5. The SciFi seeding algorithm

**Figure 5:**
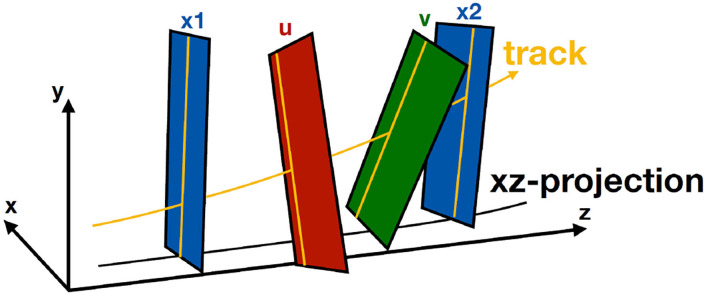
Representation of a typical T-track trajectory in the SciFi. The x-z trajectory is modeled as a parabola with a fixed cubic correction, while the y-z trajectory is a line.

Hybrid seeding, detailed in Aiola et al. (2021), is an iterative reconstruction algorithm that uses information from the SciFi subdetector of LHCb to reconstruct track segments, also called T-tracks. It exploits the four-layered structure of the SciFi stations, as shown in Figure 5, which have two vertical outer (X) layers and two inclined inner (U, V) layers, which are inclined by ±5° to determine the *y*-position of the trajectory.

This algorithm operates in iterations or cases, starting from different layers to cover for hit inefficiency. Between each iteration, hits that were used to form a good-quality track are removed from the container, which allows the loosening of tolerance windows and track quality requirements as the environment is cleaned up by the algorithm.

Each iteration can be separated into two parts: the *x* − *z* track finding and the U/V hit addition. The trajectory of a track, as shown in Figure 5, can be approximated as a parabola with a fixed cubic correction in the *x* − *z* plane and a straight line in the *y* − *z* plane. Due to the geometry of the SciFi detector, y information can only be accessed using the *x*(*z*) equation.

**Figure 6:**
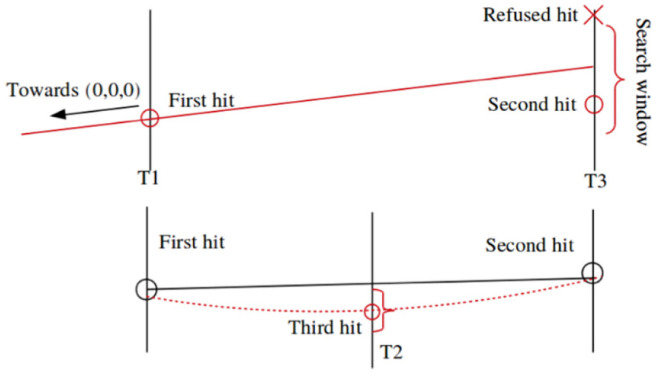
Formation of the triplets in x-z search. The position of a hit in a first layer is projected onto a second layer assuming infinite momentum, and all hits around that projection, given a momentum-dependent tolerance, are used to form 2-hit combination. A third hit is then looked for in a third layer using the estimated momentum from the 2-hits combination.

Figure 6 shows the principle of the triplet finding in the *x* − *z* plane, which is the most computationally expensive part of the algorithm. Each hit in the first considered X layer is propagated onto a second X layer, assuming the track has infinite momentum and comes from the origin. A tolerance window is opened around that projection, assuming a minimum momentum, and all hits within this window are collected. The difference between the position of the second hit and the infinite momentum projection is a measurement of the momentum, which allows to estimate the bending within SciFi and to look for the third hit. Each triplet then provides the full track equation, and remaining hits are searched for. Tracklets are then selected according to quality criteria such as χ^2^ of the fit to the trajectory and the number of hits.

Given the *x*(*z*) track equation, hits on tilted U and V layers can be converted to a *y* measurement. Considering that we expect tracks to be a straight line coming from the origin, these *y* measurements are converted to a slope measurement, and the algorithm looks for the accumulation of similar slope measurements in different U/V layers. This is called Hough transformation and clustering. Each Hough cluster which has enough hits is then tested by adding it to the *x* − *z* track and performing a global fit to the trajectory. Tracks that do not pass quality requirements on that fit are rejected. The seeds reconstructed by the Hybrid seeding are then used to form more complex tracks. They are matched with VELO segments to form Long tracks or with hits in the UT to form Downstream tracks. The remaining tracklets are then called T-tracks.

**Figure 7:**
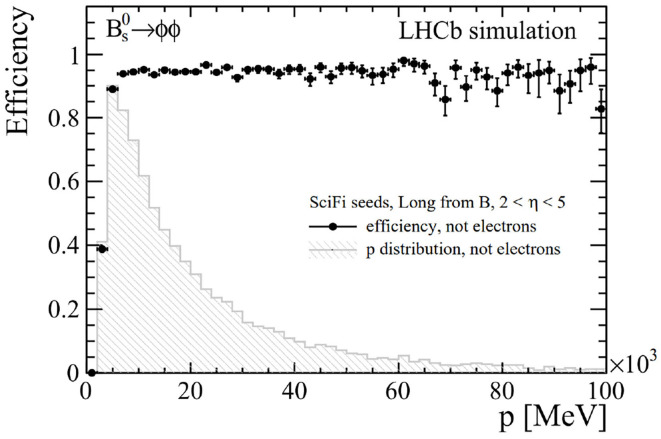
Efficiency of the Hybrid seeding reconstruction on GPUs as a function of the track momentum of Long tracks produced from *B*_*s*_ → *ϕϕ* decays [LHCb-FIGURE-2022-010].

Recently, an optimization and high-throughput configuration of this algorithm has been implemented in the new GPU architecture of the LHCb trigger system. The main features of this new seeding is the high level of parallelism and the reimplementation of the U/V hit addition as a sequential hit search, similar to that performed in the *x* − *z* part. Additionally, some quality criteria have been tightened. Figure 7 shows the preliminary physics performance of the Hybrid seeding algorithm as implemented on GPUs on Long tracks coming from the *B*_*s*_ → *ϕϕ* decay channel, where the *ϕ* meson is reconstructed from two kaons of opposite signs that traverse the SciFi detector.

### 2.6. Higgs portal to dark matter

Some models of physics beyond the Standard Model (BSM) predict the Standard Model (SM) Higgs field serving as the portal to a dark sector, which could accommodate dark matter candidates (Kachanovich, Aliaksei et al., 2020). One of the simplest and well-known models in this regard predicts the existence of a mixed state between a new scalar low-mass boson (H') and the SM Higgs (H), regulated by the mixing strength θ:


(1) $$\begin{array}{l} h=H\text{cos}\theta -H^{\prime }\text{sin}\theta \end{array}$$

**Figure 8:**
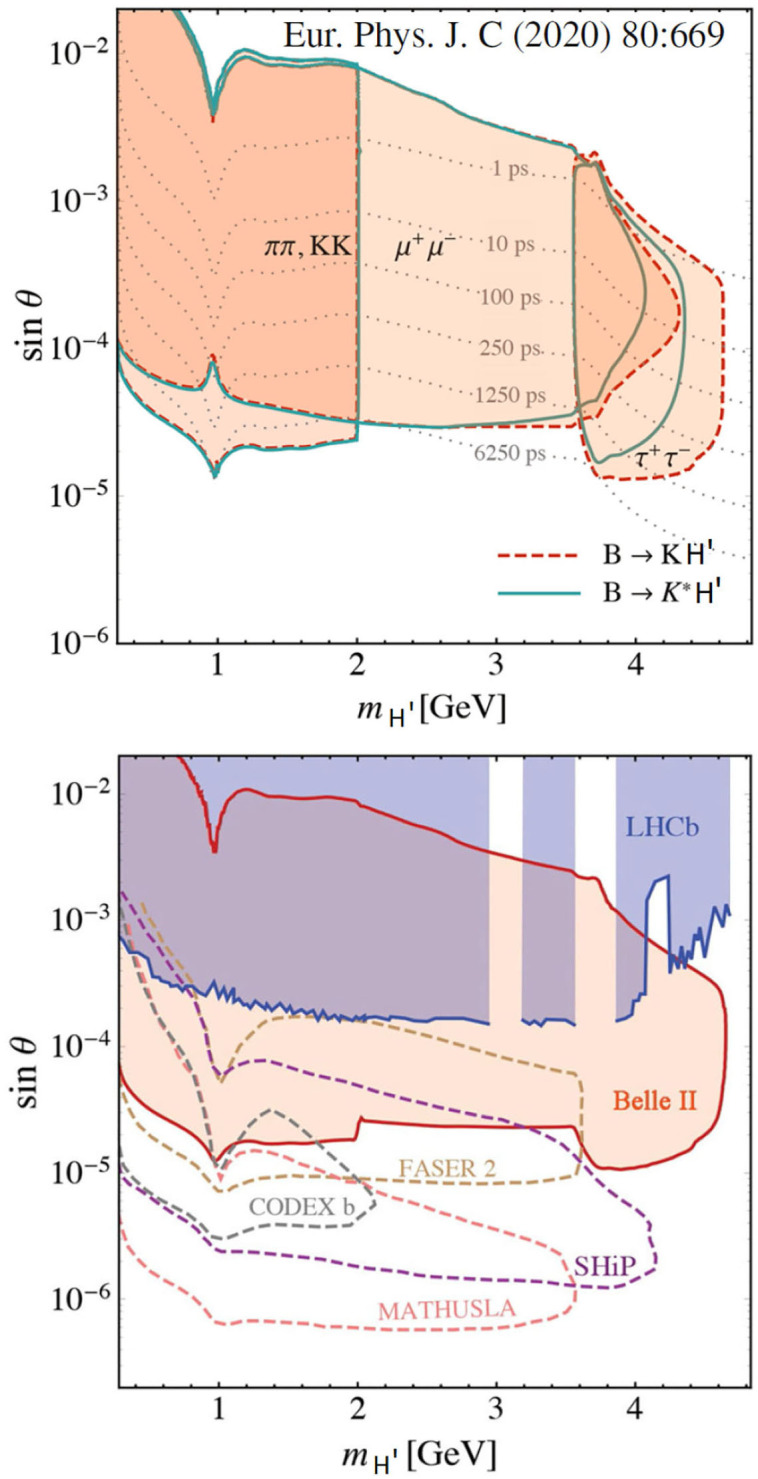
Parameter space regions for the *B* → *H*′*K* and *B* → *H*′K^*^ decay channels **(top)** and sensitivity to the H' displaced vertex by different present and future experiments **(bottom)**, (Figures adapted from Kachanovich, Aliaksei et al., 2020).

In this model, H' can be interpreted as a mediator to a dark sector of unknown mass and lifetime. This model could be validated through the experimental signature of the decay *B*→*H*′*K*, with the H' decaying into π^+^π^−^, *K*^+^*K*^−^, *μ*^+^*μ*^−^, orτ^+^τ^−^, depending on its mass. A displaced vertex could be determined, allowing to reconstruct the H' mass from the kinematics and the identification of the two decay particles. The sensitivity to this model depends nevertheless on the H' mass and lifetime, which could lead the new scalar to decay outside the detector fiducial volume. In particular, if the H' has long lifetime, the two final decay particles would not be selected by the first level of the LHCb trigger, thus escaping detection. Figure 8 shows the parameter regions and the sensitivity of different (present and future) experiments to the H' (Kachanovich, Aliaksei et al., 2020). One should note that the mixing angle is directly related to the decay width, and thus to the lifetime, with small mixing angles implying long lifetimes.

Considering the leptonic decay mode H' → *μ*^+^*μ*^−^, the decay rate can be expressed by:


(2) $$\begin{array}{l} \Gamma \left(H^{\prime }\to \ell \ell \right)=\sin^{2}\theta \frac{G_{F}m_{H^{\prime }}m_{\ell }^{2}}{4\sqrt{2}\pi }{\left(1-\frac{4m_{\ell }^{2}}{m_{H^{\prime }}^{2}}\right)}^{3/2}, \end{array}$$

where *G*_*F*_ is the Fermi constant and *m*_*l*_ the lepton mass. The Higgs lifetime can be computed:


(3) $$\begin{array}{l} \tau_{H^{\prime }}=\frac{1}{\Gamma \left(H^{\prime }\to \mu^{+}\mu^{-}\right)}. \end{array}$$

The following sections discuss the sensitivity of the LHCb experiment at the trigger level to the H' decay into the *μ*^+^*μ*^−^ final state. This decay channel binds the Higgs mass to be *m*_*H*_ > 2*m*_*μ*_ ≈ 212 MeV/c^2^. The H' mass is also constrained to $m_{H^{\prime }}<m_{B^{+}}-m_{K^{+}}\approx 4700$ MeV/c^2^.

## 3. Results

### 3.1. Sensitivity to *B*→*H*′(→ *μ*^+^*μ*^−^)*K* decays

Using the upgraded LHCb simulation, 99 MC samples of 7,000 events each are simulated from proton-proton collisions. The samples are generated using Pythia8 and assuming Run 3 beam conditions. The *B*→*H*′(→ *μ*^+^*μ*^−^)*K* decay channel is generated considering H' masses in the range of 500–4,500 MeV and lifetimes from 1 to 2,000 ps.

**Figure 9:**
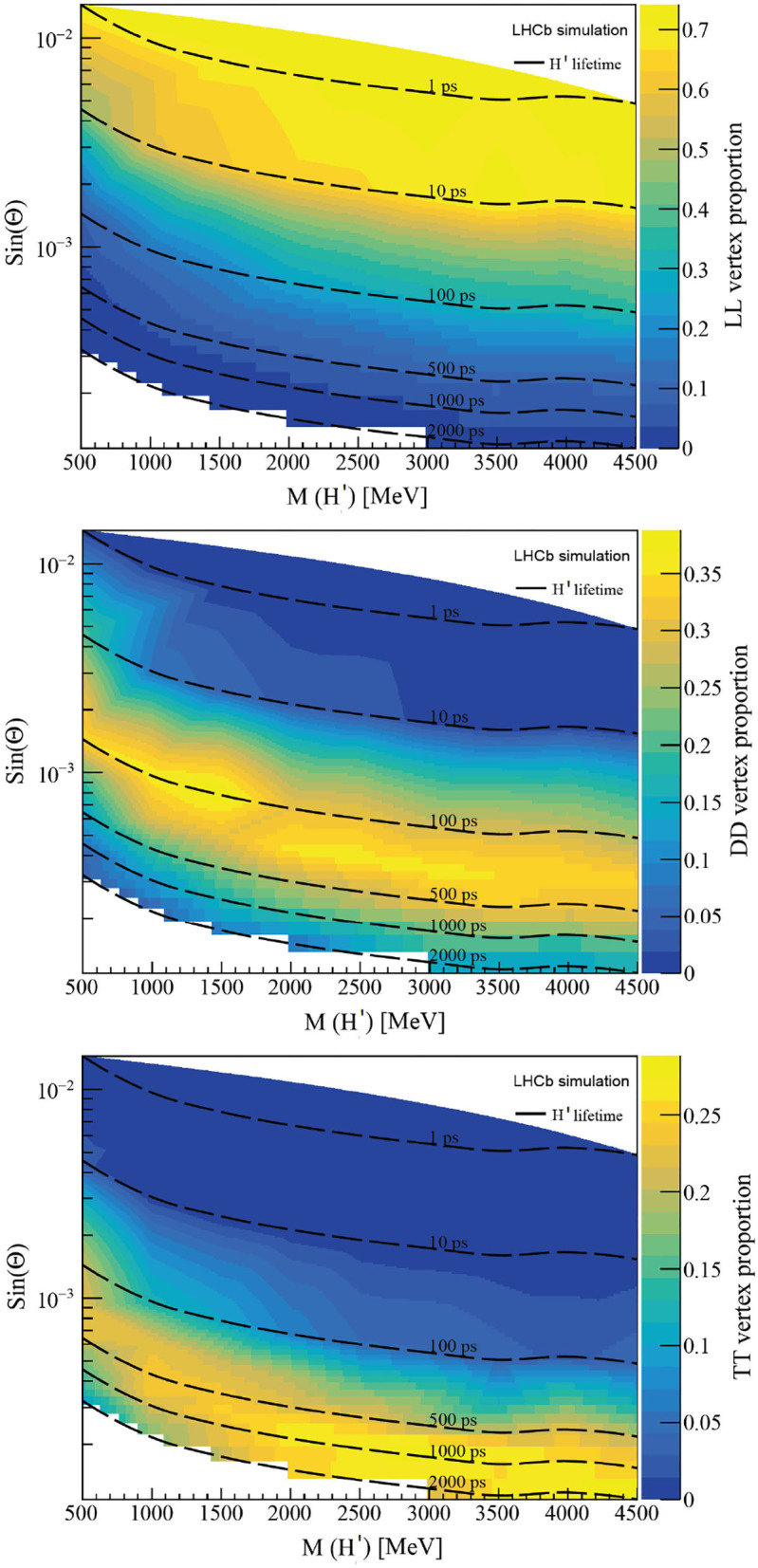
Reconstructibility of the decay vertex of the H' particle as a function of its mass and lifetime. Decay topologies are shown, from top to bottom, in the order: LL, DD, and TT, corresponding to the track types of the two muons.

The decay vertex of the H' is expected to be displaced with a dependence on these variables, and they will be labeled in the following according to the track type of the two muons (two Long tracks = LL, two Downstream tracks = DD and two T-tracks = TT). LL vertices are thus expected to be produced in the Velo detector, DD between the Velo and the UT, and TT vertices between the UT and SciFi. The reconstructibility of these vertices is defined according to the track reconstructibility criteria defined in Section 2.3, and imposing that the two muons are coming from the same vertex. Figure 9 shows the reconstructibility of the decay vertex of the H' particle as a function of its mass and lifetime. For lifetimes below 10 ps, a large proportion of LL vertex topologies is found, as expected, where the H' decays in the VELO acceptance and both muons can be reconstructed as Long tracks. Nevertheless, for H' lifetimes larger than 100ps (and small-mixing angle), most of the decays are produced downstream the VELO, resulting in a large proportion of the DD and TT topologies. These fractions are very similar in the case that the H' decays into two hadrons^1^.

**Figure 10:**
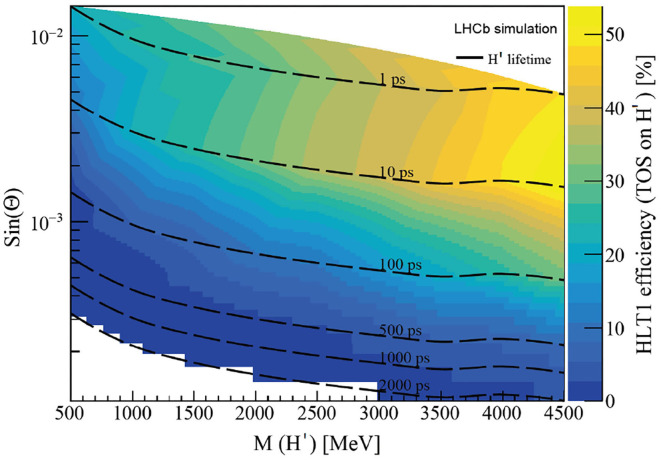
Proportion of events triggered by the HLT1 decision on the H' decay products (Trigger on Signal, TOS).

Figure 10 shows the LHCb HLT1 effect when triggering on the H' decay products (Trigger on Signal, TOS). Since only Long tracks are reconstructed at the HLT1 level, a high inefficiency can be observed for large H' lifetimes, going down 10% for lifetimes larger than 500ps. A loss in sensitivity for small H' masses is also observed, and since the H' is experiencing larger boosts, muons, are escaping from detection in the VELO.

### 3.2. Study of long-lived SM particles: Λ^0^ and $K_{\text{S}}^{0}$

**Figure 11:**
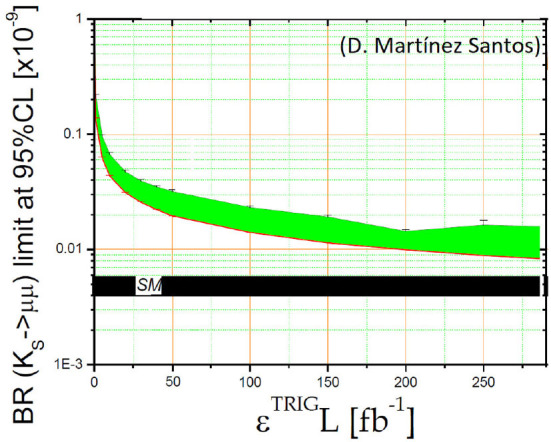
Sensitivity (green band) to the $K_{\text{S}}^{0}$ → *μ*^+^*μ*^−^ branching fraction as function of the trigger efficiency × luminosity. The SM prediction is shown in black (Santos, 2022).

Some strange SM particles, such as $K_{\text{S}}^{0}$ and Λ^0^ baryons, also have long lifetimes of the order of 100ps. These particles are reconstructed in many physics analysis to measure observables which can be sensitive to new physics models. One example is the rare $\Lambda_{b}\to \Lambda^{0}\gamma$ decay, where the Λ^0^ baryon decays into a proton and a pion. Measurements of the branching fraction and the angular distribution of the decay particles are sensitive to models with non-standard right-handed currents (Aaij et al., 2019, 2022; Garćıa Mart́ın et al., 2019). Another example is the very rare $K_{\text{S}}^{0}$ → *μ*^+^*μ*^−^ decay, very suppressed in SM, not observed experimentally at present, which is sensitive to different BSM scenarios such as SUSY (Zhu, 2012) or presence of leptoquarks (Bobeth and Buras, 2018). Figure 11 shows the sensitivity to the $K_{\text{S}}^{0}$ → *μ*^+^*μ*^−^ decay channel (in terms of branching fraction limit) as a function of the product of the trigger efficiency and luminosity.

**Figure 12:**
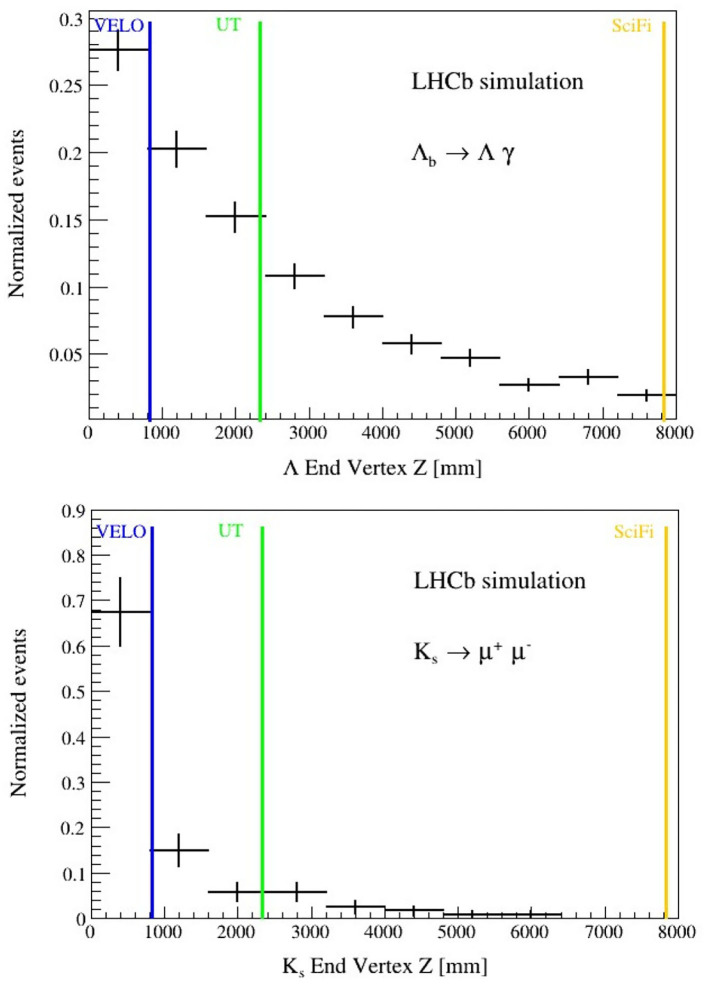
Λ^0^ **(top)** and $K_{\text{S}}^{0}$ **(bottom)** reconstructible candidates as function of the decay vertex position. The Λ^0^ candidates decay into a pion and a proton. The $K_{\text{S}}^{0}$ candidates decay into two muons of opposite charges. Vertical color lines indicate the positions of the Velo, UT, and SciFi detectors in the Z-axis.

Using 10,000 simulated events, the HLT1 trigger effect has been studied on these two decay channels involving Λ^0^ and $K_{\text{S}}^{0}$ particles. Figure 12 shows the normalized number of reconstructible events (LL+DD+TT) as a function of the end decay vertex of the Λ^0^ and of the $K_{\text{S}}^{0}$.

The relative proportions of track types are 12% (LL), 51% (DD), and 37% (TT) for the $\Lambda_{b}\to \Lambda^{0}\gamma$ decay channel. For the case of prompt $K_{\text{S}}^{0}$, the amount of reconstructible tracks is 46% (LL), 38% (DD), and 16% (TT), One should notice that a large amount of decays occurs at the end of the VELO and they do not let enough hits to be reconstructed as Long tracks, being reconstructed thus in the DD category. That is, the amount of DD+TT tracks is about 88% for the $\Lambda_{b}\to \Lambda^{0}\gamma$ decay channel and 54% for prompt $K_{\text{S}}^{0}$.

One can apply the HLT1 conditions on the reconstructible events, and in particular, to check how many events are selected by inclusive trigger lines, such as the OneTrackMVA or TwoTrackMVA. They require tracks with minimum transverse momentum and minimum Impact Parameter (IP) significance with respect to the Primary Vertex, and for the latter, they form a vertex with minimum requirements. In the case of $\Lambda_{b}\to \Lambda^{0}\gamma$, the HLT1 signal efficiency for the proton and pion coming from the Λ^0^ is found to be less than 10%. In the case of $K_{\text{S}}^{0}$, the HLT1 efficiency on the muons, adding some inclusive muon lines and dedicated lines for $K_{\text{S}}^{0}$ selection, is less than 25%. We normalize the efficiency to the sum of LL+DD+TT reconstructible events. The $K_{\text{S}}^{0}$ candidates in this study are prompt, produced at the interaction point. In the case that $K_{\text{S}}^{0}$ candidates are coming from the decays of b or c-hadrons, the amount of reconstructible LL candidates is expected to decrease, thus increasing the HLT1 inefficiency.

## 4. Prospects

A downstream algorithm, making use of the high performance of the Seeding algorithm described earlier, is currently being developed within the LHCb-RTA Collaboration. Preliminary studies showed that, in the case that DD tracks could be reconstructed and selected at the HLT1 stage, efficiencies for LLPs could increase to 60% or more. A lot of effort is also being devoted to the development of inclusive trigger lines based on SciFi seeds, even if the high occupancy and track combinatorics in this detector makes it quite challenging in terms of allowed rates. The use of machine learning algorithms is also being investigated, which could be applied at an early stage to improve the T-track classification. With the new HLT1 framework based on the GPU architectures, this task seems feasible, now or in a near future, by using faster and better performing GPU cards.

## 5. Conclusion

In summary, during the past years, the LHCb detector has undergone a major upgrade, which will allow the data for Run 3 to be taken at an averaged proton-proton collision rate of 30 MHz. The first trigger stage of the LHCb will be based for the first time entirely on GPU cards following the Allen framework, a complete high-throughput trigger algorithm design. This will largely increase the capabilities of the trigger system for selecting the data of interest. Tracking reconstruction at early stages of the data chain is becoming an arduous task due to the increase of the output data. This is even more challenging if the reconstruction involves particle trajectories that do not leave signals in the first tracker of the LHCb detector, hitting only the UT and/or the SciFi, which are bigger detectors with high occupancy. Even if the main program of the LHCb experiment is based on the reconstruction of b- and c-hadron decay particles, which are usually Long tracks, there is an important physics case for Downstream and T-tracks to be participating in the first stage of the trigger. The trigger efficiency for long-lived particles is at present very low. This is true for both the cases of BSM and in the Standard Model formalism. In this study, we studied the impact of the HLT1 on a model based on a Higgs portal to a dark sector, where the Higgs can have several masses and lifetimes. For long lifetimes and small masses of the Higgs, the HLT1 inefficiency is found to be quite large. We also tested how the HLT1 decisions affect Λ^0^ and $K_{\text{S}}^{0}$ decays, as observed in many Standard Model processes. For these cases, the selected events are one-fourth or less of the possible reconstructible candidates. To improve the HLT1 efficiency, an algorithm is being developed in the Allen framework, which makes the use of the SciFi seeds to reconstruct trajectories of particles with long lifetimes. The inclusion of this algorithm will allow to largely increase the efficiency for Λ^0^ and $K_{\text{S}}^{0}$ and would open a large window for the searches of many particles beyond the Standard Model.

## 6. LHCb-RTA collaboration

The authors of this article want to acknowledge the contribution of Christina Agapopoulou and Lorenzo Pica to the development of the seeding and matching trigger algorithms on GPUs. The contribution of Renato Quagliani has also been decisive for the development of the algorithm. We acknowledge the theoretical discussions with Xabier Cid Vidal. The study of Fernando Martinez Vidal and Nicola Neri, as well as the Università di Milano LHCb group on the usability of T-tracks in LHCb, is also very relevant in this context. Similarly, the study of Giovanni Punzi and the LHCb group of the Università di Pisa related to other hardware architectures will be crucial for this study in the near future. We also express our gratitude to our colleagues of the LHCb Collaboration, in particular to the LHCb's computing, simulation, DPA, and online projects. This study has been developed in the framework of the RTA project, so we thank our colleagues of RTA for their support and dedication.

## Data availability statement

The datasets used in this work have been produced and analyzed inside the LHCb computing framework and are subject to the LHCb agreements and rules. They could be made available upon request, ensuring compliance with privacy agreements and other requirements.

## Author contributions

LC, LH, AH, BJ, and JZ worked on the Hybrid seeding algorithm and achieved its implementation on GPUs. CVS developed the framework for MC simulations BSM. AO, LH, and DM performed the sensitivity studies including different decay channels and physics models. JZ also contributed to get the track reconstructibility for SM samples. IS worked on the reconstruction of T-tracks from the SciFi seeds and validated their use for physics analyses. AO is the main editor of the paper. All authors have contributed in the edition of the paper and the creation of the figures.

## Funding

This work is supported and has been developed in the framework of the RTA project inside the LHCb collaboration. This work has been partially funded by the CERN and the MICINN national agency in Spain. AH is supported by the NSF award 1904160. DM is supported by the CONEXION AIHUB-CSIC. LC is funded by the ERC Refs: 724777-RECEPT and 714536-PRECISION, as well as the PUNCH4NFDI consortium supported by the DFG fund NFDI 39/1.

## Conflict of interest

The authors declare that the research was conducted in the absence of any commercial or financial relationships that could be construed as a potential conflict of interest.

## Publisher's note

All claims expressed in this article are solely those of the authors and do not necessarily represent those of their affiliated organizations, or those of the publisher, the editors and the reviewers. Any product that may be evaluated in this article, or claim that may be made by its manufacturer, is not guaranteed or endorsed by the publisher.

## Footnotes

^1^One should note that the tracking reconstruction at this level does not include particle identification.
